# Evaluation of Agricultural Traits, Phytochemical Contents, and Antioxidant Activities in the Leaves of Amaranth Accessions of Nine Species

**DOI:** 10.3390/plants11131758

**Published:** 2022-07-01

**Authors:** Weilan Li, Eunae Yoo, SooKyeong Lee, Hyung Jun Noh, So Jeong Hwang, Kebede Taye Desta, Gi-An Lee

**Affiliations:** 1 National Agrobiodiversity Center, National Institute of Agricultural Sciences, Rural Development Administration, Jeonju 54874, Korea; weilan@korea.kr (W.L.); eung77@korea.kr (E.Y.); xsanta7@korea.kr (S.L.); ghkdthwjd7@korea.kr (S.J.H.); kebedetdesta@korea.kr (K.T.D.); 2 International Technology Cooperation Center, Rural Development Administration, Jeonju 54875, Korea; jumpspace@korea.kr; 3 Department of Applied Chemistry, Adama Science and Technology University, Adama 1888, Ethiopia

**Keywords:** amaranth, agricultural traits, phytochemical, antioxidant activity

## Abstract

*Amaranthus* species are widely cultivated as dietary crops and are promising sources of phytochemical compounds with antioxidant properties. To explore *Amaranthus* as a potential medical resource, 289 accessions (nine species) were cultivated, and their agricultural characteristics, total phenolic content (TPC), rutin contents, and antioxidant activities [2,2-diphenyl-1-picrylhydrazyl (DPPH) and 2,2′-azino-bis-(3-ethylbenzothiazoline-6-sulfonic acid) (ABTS)] were studied. Wide variations in agricultural traits, phytochemical contents, and antioxidant activities were observed between the accessions and across species. The effects of agricultural traits were evaluated, and the results indicated that yellow-flowered amaranth genotypes could be important because of their high values of TPC, rutin contents, DPPH, and ABTS. In addition, leaf length, days until 50% flowering, days until 50% heading and days until maturity, showed positive correlations with TPC, rutin contents, DPPH, and ABTS. The whole dataset was subjected to principal component analysis, and distinctive aggregation was observed across the *Amaranthus* species. In total, 289 accessions were clustered into three groups, and seven genotypes were determined as being good medical resources due to their high phytochemical content and antioxidant activities. Our findings provide important information for the development of new varieties with high phytochemical contents and high levels of antioxidant activity.

## 1. Introduction

Amaranth (*Amaranthus* spp. L.), which comprises about 70 species, is an annual plant in the family *Amaranthaceae*. As a superfood, it was named “the grain of God”, and it is widely distributed and cultivated in tropical and subtropical areas around the world [1,2]. Amaranth is a promising food crop in arid regions because of its rapid growth and resistance to stressful environments [3,4]. The morphological diversity of *Amaranthus* is remarkable, and there are obvious morphological differences within species. The plants can grow to a height of 1–2.5 m, and variations have been reported in the sharpness and color of leaves, flowers, stems, and seed coats [5]. A previous study also reported wide variations in other morphological traits, including stem diameter, leaf number, and leaf thickness, in weedy types of Indonesian amaranth [6]. Such morphological differences illustrate the genotypic diversity and high variability of phenotypic features at the genetic level; therefore, investigating genetic variations, and relationships between agricultural traits and metabolite levels, could provide useful data for breeding new varieties that are both rich in metabolites and that possess the desired morphological appearance [6,7].

*Amaranthus* species are domesticated and cultured for use as a vegetable, grain, fodder, an ornamental plant, and in traditional medicine [8,9]. Among the different tissues, those that comprise amaranth leaves and stems are of great interest because of their high nutritional value. They have high amounts of iron, copper, calcium, and other minerals, as well as carotene, vitamin C, essential amino acids, phenolic compounds, flavonoids, lignins, and hydroxybenzoic acids, among others [10,11,12,13,14,15]. Moreover, extracts from various *Amaranthus* species have been used in therapies for urinary infections, respiratory disorders, pain, cardiovascular diseases, hypertension, tumors, and diabetes [16,17]. Studies have demonstrated that polyphenolic compounds, which are among the most widely used plant chemicals, have powerful antioxidant effects. They have been studied for suppressing inflammation, and for treating dementia and atherosclerosis [18,19,20]. Several *Amaranthus* species are rich sources of polyphenolic compounds, and they are of increasing interest to scientists due to their potential for preserving human health. Among the different classes of polyphenols, flavonoids are considered effective for inhibiting age-related diseases. Rutin, also known as rutoside, quercetin-3-rutinoside, and sophorin, is one of the most common flavonoids in plants, and is effective for colorectal carcinogenesis therapy [21]. From a dietary perspective, the rutin content of amaranth is an important factor, and high levels of this compound are important for the prevention of diseases caused by modern society [22,23]. Due to their broad range of pharmacological effects and metabolite contents, *Amaranthus* species are becoming popular with many consumers [24].

Many scientists have studied the biochemical contents and antioxidant activities of *Amaranthus* genotypes [8,24,25,26,27,28,29]; however, few have investigated the correlations between agricultural traits, phytochemicals, and antioxidant activities [6,10,15,30,31,32]. To obtain a thorough understanding of such associations, studies involving large numbers of amaranth genotypes are required. In this study, 289 amaranth accessions (nine species) were obtained and cultivated under uniform field conditions. Next, the associations between agricultural traits, phytochemical contents, and antioxidant activities were investigated. The results of this study could provide important knowledge for the propagation of amaranth, and it could help breeding programs develop new amaranth varieties with high metabolite contents and strong antioxidant activities. 

## 2. Materials and Methods

### 2.1. Reagents and Chemicals

The chemicals and reagents used in this study, including the Folin–Ciocalteu phenol reagent, 2,2-diphenyl-1-picrylhydrazyl (DPPH), 2,2′-azino-bis-(3-ethylbenzothiazoline-6-sulfonic acid) (ABTS), rutin, sodium carbonate, ethanol, gallic acid, standard Trolox, and methanol, were purchased from Sigma Aldrich (St. Louis, MO, USA). All chemicals were of analytical grade and used without further purification.

### 2.2. Amaranth Cultivation and Sample Preparation

The seeds of 289 amaranth genotypes of nine species, including *A. cruentus* (n = 22), *A. hybridus* (n = 21), *A. hypochondriacus* (n = 77), *A. powellii* (n = 7), *A. quitensis* (n = 8), *A. spinosus* (n = 13), and *A. tricolor* (n = 77), were obtained from the gene bank of the National Agrobiodiversity Center (NAC), Rural Development Administration (RDA), Jeonju, South Korea. The seeds, which originated from more than 40 countries, were seeded and cultivated on a research farm at the NAC in 2021. 

Initially, amaranth leaves were dried in an oven (40 °C) and ground into a fine powder. Crude extraction was performed following a previously described method, with some modifications [33]. Briefly, 2 g of each sample (in triplicate) was mixed with 20 mL of 75% ethanol, and the mixture was processed with an accelerated solvent extractor (ASE) (ASE-350; Dionex, Sunnyvale, CA, USA) using nitrogen gas for 15 min. The pressure and temperature were set at 1200 psi and 70 °C, respectively. After extraction, the crude extracts were transferred separately to a new 50 mL conical tube and they were concentrated using a vacuum concentrator (HT-6; Genevac, Ipswich, UK) at 40 °C for 10 h. 

### 2.3. Agricultural Traits

Agricultural traits were measured according to a previously described method [6]. Leaf length (LL), leaf width (LW), panicle length (PL), and panicle width (PW) were measured using a slide caliper. Values are represented as the average of five plants for each accession. The leaf color, flower color, seed coat color, days until 50% flowering (FD), days until 50% heading (HD), and days until maturity (MD) of each accession were recorded during field inspections. 

### 2.4. Determination of Total Phenolic Content

The total phenolic content (TPC) was determined using the Folin–Ciocalteu colorimetric method, as described by Waterhouse [34], and subsequently modified by Assefa et al. [33], with gallic acid used as a standard. The dried extracts were dissolved in 75% ethanol, using appropriate concentrations, filtered through a 0.45-µm filter using a needleless syringe, and readied for TPC analysis. The Folin–Ciocalteu reagent (100 µL) was added to each sample or standard (100 µL), and they were incubated at room temperature for 3 min. Next, 100 µL of 2% Na_2_CO_3_ was added to each mixture, followed by an incubation period in the dark at room temperature for 30 min for color development. The absorbance of the solutions was measured using an Eon Microplate Spectrophotometer (Bio-Tek, Inc., Winooski, VT, USA) at 750 nm, and 75% ethanol was used as a blank specimen. TPC was calculated based on the standard curves which were prepared using various concentrations of the gallic acid standard. The results are presented as μg gallic acid equivalent per mg sample (μg·GAE/mg) from triplicate measurements. 

### 2.5. Determination of Rutin Content

Rutin content was determined using the method by Tundis et al. [35], with some modifications. The obtained dried extract was dissolved in 80% methanol, using appropriate concentrations, and filtered through a 0.45-µm filter. It was identified by using ultra-high-performance liquid chromatography (UHPLC) with a Cortecs C18 (1.6 µm, 2.1 × 150 mm) column. During the analysis, a solvent system composed of solvent A (1% formic acid in water) and solvent B (1% formic acid in acetonitrile) was used as the mobile phase at a flow rate of 0.2 mL/min, and the target was detected at 350 nm. Gradient elution of the organic modifier was conducted using the following schemes: 0 min, 2% B; 15 min, 4% B; 30 min, 20% B; 75 min, 50% B; 85 min, 2% B. The concentration of rutin in amaranth leaves was calculated with a standard curve that was prepared using various concentrations of the pure rutin standard. The equation and correlation coefficient of the standard curve was y = 30.075 x + 83.441, R^2^ = 0.9992 (y is the peak area and x is rutin concentration).

### 2.6. Antioxidant Capacity Assay

Antioxidant capacity was assessed using the method by Sarker et al. [36], with some modifications. For the DPPH radical scavenging assay, 100 µL of each sample extract, dissolved in 75% ethanol, using appropriate concentrations, and filtered through a 0.45-µm filter, was mixed with 150 µL of 150 µM anhydrous ethanol, followed by a 30 min incubation period in the dark at 25 °C. The DPPH radical scavenging activity was evaluated by measuring the absorbance at 517 nm using a spectrophotometer (NanoQuant Plate; Tecan, Männedorf, Switzerland). During the ABTS assay, a working solution was initially prepared by mixing equal volumes of 7 mM ABTS solution and 2.45 mM potassium persulfate stock solution, followed by storage in the dark for 12 h at 4 °C. Next, 190 µL of ABTS working solution was added to 10 µL of each sample extract. The mixtures were kept in the dark for 30 min, followed by an absorbance measurement at 734 nm using a spectrophotometer (NanoQuant Plate; Tecan). In each case, 75% ethanol was used as a blank. The DPPH and ABTS radical scavenging activities were calculated using the following equation:

Antioxidant activity (%) = [(A_blank_ − A_sample_)/(A_blank_)] × 100

where A_sample_ is the absorbance of the test extract and A_blank_ is the absorbance of the blank sample (100 µL 75% ethanol (DPPH), 10 μL 75% ethanol (ABTS) instead of extract). Trolox was used as the standard, and the results are expressed in µg Trolox equivalent per mg (µg·TE/mg) of the dried sample.

### 2.7. Statistical Analysis

All of the experiments in this study were conducted in triplicate, and the data were subjected to analysis of variance (ANOVA) using R software (version 4.1.2; R Development Core Team, Vienna, Austria). TPC, rutin content, and DPPH radical and ABTS radical scavenging activities are reported as the mean ± standard deviation (SD). Quantitative measures of agricultural traits are reported as the average values obtained from five amaranth plants. Principal component analysis (PCA), hierarchical clustering principal component (HCPC) analysis, and partial least squares discriminant analysis (PLS-DA) were also performed using R software (version 4.1.2) [37,38,39]. 

## 3. Results and Discussion

### 3.1. Agricultural Traits

#### 3.1.1. General

**Figure 1:**
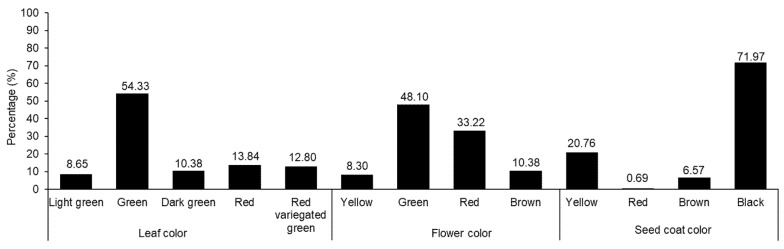
Percentage distributions of amaranth accessions based on the morphological characters of leaf, flower, and seed coat colors.

The variations in agricultural traits, including LL, LW, PL, PW, FD, HD, MD, leaf color, flower color, and seed coat color, were recorded and are presented in Table 1 and Figure 1. In each case, wide diversity was observed. Previous studies have also shown wide variations in agricultural traits among *Amaranthus* accessions [5,7,10,40]. The LL and LW values ranged from 6.84 to 32.16 and 3.00 to 21.33 cm, respectively. Likewise, PL and PW ranged from 0.66 to 29.10 and 0.50 to 6.16 cm, respectively. The FD, HD, and MD values were in the ranges of 35–108, 45–125, and 54–162 days, respectively. Compared with the results of previous studies, our results showed wider variations in some agronomic traits. For instance, Akaneme and Ani reported the LL and LW values as being in the ranges of 13.63–33.15 and 8.47–14.76 cm, respectively, and the FD value was in the range of 41.00–66.00 days [41]. Gerrano et al. recorded PL values from 17.44 to 44.57 cm when observing *Amaranthus* genetic resources from South Africa [42]. Variations were also observed in leaf, flower, and seed coat colors. Green was the predominant leaf color (54.33%), followed by red (13.84%). Green was also the main color of the flower (48.10%), followed by red (33.22%). Most of the accessions had black seeds (91.97%), followed by yellow seeds (20.76%), although two accessions produced red seeds (0.69%). 

#### 3.1.2. Variations of Agricultural Traits According to Species

The variations of agricultural traits in the nine *Amaranthus* species are shown in Table 2. Significant differences were observed between the phenotypic traits of the *Amaranthus* species. *A. cruentus* exhibited the highest average LL (25.65 cm) and LW (13.30 cm), followed by *A. caudatus* (22.05 and 12.75 cm, respectively), whereas *A. spinosus* had the lowest average LL (10.30 cm) and LW (5.53 cm) values. In addition, *A. cruentus* exhibited the highest average PW (1.64 cm). Andini et al. also reported that *A. cruentus* exhibited the largest leaf size, followed by *A. caudatus*, among the *Amaranthus* genetic resources in Indonesia [6]. In contrast, *A. spinosus* developed the highest average PL (15.48 cm) and *A. caudatus* displayed the lowest average PL (4.13 cm). It was well studied in rice that large leaf area, length, and width promote panicle development [43]. Furthermore, in another study of amaranth, LL showed a positive association with PL [44]. In the present study, *A. spinosus* had the lowest average LL and LW, but the highest average PL. This could be a special characteristic of *A. spinosus*. Moreover, *A. caudatus* displayed the longest average FD (60.74 days) and HD (74.19 days), and *A. spinosus* exhibited the shortest average FD (40.69 days), HD (59.46 days), and MD (79.15 days). In conclusion, both *A. cruentus* and *A. spinosus* showed genetic characteristics that are advantageous for breeding. *A. cruentus* could be a good donor for developing new vegetable varieties because of its larger LL and LW, and *A. spinosus* could contribute to the development of new grain varieties because of its higher PL and shorter MD. 

### 3.2. Phytochemical Contents and Antioxidant Activities

#### 3.2.1. General

The amaranth accessions showed significant variations in TPC, rutin content, and antioxidant activities (Table 1). TPC ranged from 159.62 to 958.19 µg·GAE/mg·DE, with a mean value of 456.05 µg·GAE/mg·DE. TPC had a wider range and higher average value than in other studies [15,45,46]. The rutin content was low, and varied widely among the genotypes from 0.12 to 42.30 mg/g, with an average value of 10.06 mg/g. Similarly, Li et al. [45] reported a rutin content of 333.25 ± 10.87 mg/100 g dry weight in *A. hypochondriacus* and 264.21 ± 5.57 mg/100 g dry weight in *A. caudatus*. Sarker and Oba reported that the rutin content ranged from 17.29 to 46.56 µg g^−1^ FW in leafy vegetable amaranth [47]. Moreover, in another study, the rutin content also varied remarkably among amaranth genotypes according to species and development stage [22]. The antioxidant activities also varied significantly among the amaranth genotypes. The DPPH radical scavenging activity ranged from 1.03 to 49.22 µg·TE/mg·DE, with a mean value of 20.24 µg·TE/mg·DE. The ABTS radical scavenging activity was in the range of 75.20–449.61 µg·TE/mg·DE, with a mean value of 200.73 µg·TE/mg·DE. Compared with previous studies, the DPPH radical and ABTS radical scavenging activity values recorded in the present study showed wider variations [15,48,49]. This could be attributed to the large number of genotypes considered in the present study. Additionally, studies have shown that differences in experimental methods and genotypes also affect the metabolite levels and antioxidant activities of various plants [50]. 

#### 3.2.2. Association of Agronomic Characters with Metabolite Contents and Antioxidant Activities

**Figure 2:**
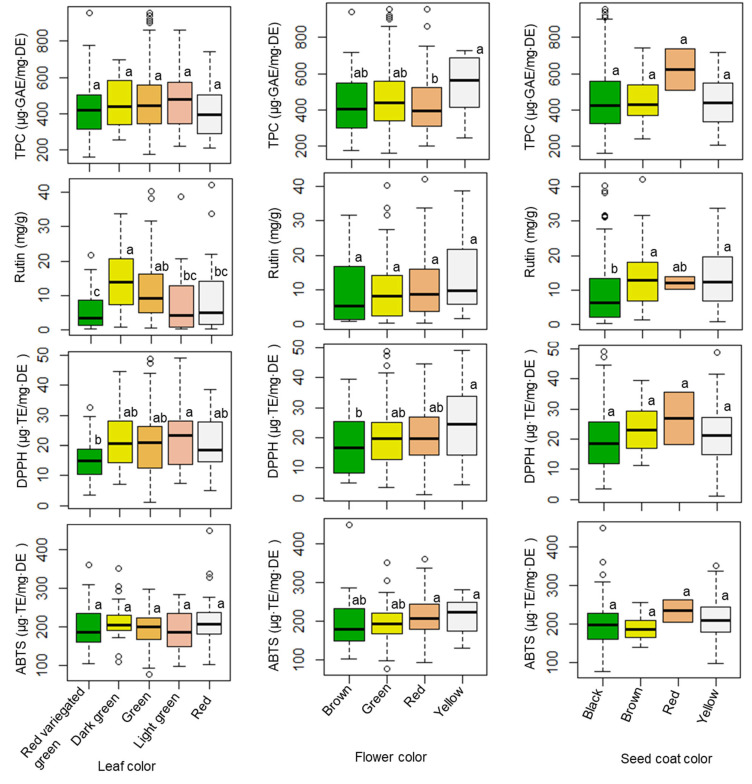
Variations of TPC, rutin, DPPH, and ABTS according to leaf, flower, and seed coat colors. Different letters indicate significant differences between groups (*p* < 0.05). TPC = total phenolic content, DPPH = DPPH radical scavenging activity, ABTS = ABTS radical scavenging activity.

The influences of seed coat color, flower color, and leaf color on TPC levels, rutin content, and antioxidant activities were also investigated, and the results are provided in Figure 2 and Table S1. The TPC value did not show a significant difference among genotypes possessing different leaf or seed coat colors; however, the TPC level varied significantly (*p* < 0.05) between genotypes with different flower colors. Amaranth accessions that developed a yellow flower color had the highest average TPC level (533.18 µg·GAE/mg·DE). Regarding rutin content, differences in both leaf and seed coat color highlighted remarkable and significant variations (*p* < 0.001), unlike flower color (*p* < 0.1). Accessions that had dark green leaves, yellow flowers, and brown seed coats had the highest average rutin content (14.22, 14.08, and 14.25 mg/g, respectively). Once again, antioxidant activities did not differ significantly according to seed coat color. Unlike these results, both the DPPH radical and ABTS radical scavenging activities differed by flower color. Leaf color highlighted significant differences in DPPH radical scavenging activity, but not ABTS radical scavenging activity. 

Several studies have investigated the phytochemical and antioxidant activities of different *Amaranthus* species and genotypes, as well as in the different tissues of the sprout, flower, leaf, stalk, and seed [15,45,50,51]; however, few studies have investigated the associations between agricultural traits, phytochemical content, and antioxidant activities across a large population of amaranth species. This study evaluated the associations between agricultural traits, phytochemical content, and antioxidant activities in amaranth leaves using a large number of amaranth genotypes from various species. More specifically, our results revealed the relationships between agricultural traits (leaf, flower, and seed coat colors) and the phytochemical and antioxidant activities of amaranth leaves. Overall, seed coat color might not be a useful parameter for discriminating between amaranth genotypes because it was not significantly associated with the TPC level or DPPH and ABTS radical scavenging activities; however, amaranth accessions with a red seed coat color could be important resources, as they displayed the highest TPC level and strongest antioxidant activities. Nevertheless, in this study, only two accessions (IT262653 and IT262667) had red seed coats. Hence, further studies of such genotypes are highly recommended. In addition, the average TPC level, rutin content, and DPPH and ABTS radical scavenging activities ranked highest in terms of accessions that developed a yellow flower color. These observations indicate that such genotypes could be useful for breeding improved cultivars. Moreover, the average level of rutin content (10.39 mg/g) and average ABTS radical scavenging activity (212.27 µg·TE/mg·DE) in red-flowered accessions were not significantly different from the highest levels of rutin content (14.08 mg/g) and ABTS radical scavenging activity (214.36 µg·TE/mg·DE) observed in yellow-flowered accessions. This indicates that accessions with red flowers could be additional sources of antioxidants. 

#### 3.2.3. Variations of Metabolite Content and Antioxidant Activities between Species

The distributions of TPC, rutin content, and DPPH and ABTS radical scavenging activities in the 289 accessions are presented in Table 3. Among the nine *Amaranthus* species, the highest average TPC level was recorded in the *A. caudatus* accessions (494.27 µg·GAE/mg·DE), followed by the *A. hypochondriacus* accessions (473.84 µg·GAE/mg·DE). The lowest average TPC level (277.18 µg·GAE/mg·DE) was found in the *A. powellii* accessions (*p* < 0.05); however, other studies found a higher TPC level in *A. hypochondriacus* than *A. caudatus* [45,49], and in *A. hybridus* than *A. hypochondriacus* [49]. Our study evaluated a larger number of amaranth species genotypes; thus, as a result, the data showed a wider variation compared with other studies. A significant variation in rutin content was also observed among the nine species (*p* < 0.001). *A. cruentus* accessions displayed the highest average rutin content (17.88 mg/g), followed by *A. hybridus* (12.71 mg/g). *A. spinosus* exhibited the lowest average rutin content (3.74 mg/g). Similarly, Kalinova and Dadakova, who also evaluated the rutin content among *Amaranthus* species, concluded that *A. hybridus* and *A. cruentus* were the best sources of rutin [22]. A comparison of DPPH and ABTS radical scavenging activities across the nine *Amaranthus* species also revealed significant variation (*p* < 0.001). *A. cruentus* had the highest average level of DPPH radical scavenging activity (24.34 µg·TE/mg·DE), and *A. spinosus* had the lowest average level of DPPH radical scavenging activity (11.62 µg·TE/mg·DE). The highest average level of ABTS radical scavenging activity was exhibited by *A. hybridus* (236.97 µg·TE/mg·DE), and the lowest average value was found in *A. spinosus* (160.84 µg·TE/mg·DE). Likewise, Bang et al. [49] compared the antioxidant activities across *Amaranthus* species. *A. hybridus* exhibited the highest DPPH and ABTS values among *Amaranthus* species in 2018; however, *A. dubius* displayed the highest levels in 2019. Our results differed slightly from the results of previous studies. Despite the different environmental conditions, genetic variation could also explain the antioxidant activity differences. 

### 3.3. Pearson’s Correlation, PCA and PLS-DA

The associations between TPC, rutin content, antioxidant activities, and agricultural traits were calculated using Pearson’s correlation analysis. The correlation coefficients (r) and their level of significance are given in Table 4. TPC showed significant positive correlations with rutin content (r = 0.25) and DPPH radical scavenging activity (r = 0.22). Likewise, significant positive associations were observed between rutin content, DPPH radical scavenging activity (r = 0.68), and ABTS radical scavenging activity (r = 0.45). Moreover, the correlation between the two antioxidant activities was strong and significant (r = 0.44). Our results corroborated previous studies that also reported positive associations between antioxidant activities and phytochemical content [15,45,48,49]. Interestingly, regarding the morphological characteristics, LL exhibited a significant positive correlation with rutin content (r = 0.40); hence, this could be an important parameter for the development of high rutin content genotypes. The relationships between the morphological characteristics were also investigated. LL showed significant positive correlations between LW, FD, HD, and MD. Furthermore, FD, HD, and MD showed significant positive correlations with each other. Similarly, other studies obtained positive correlations between LL and LW, as well as FD and MD [44,52].

**Figure 3:**
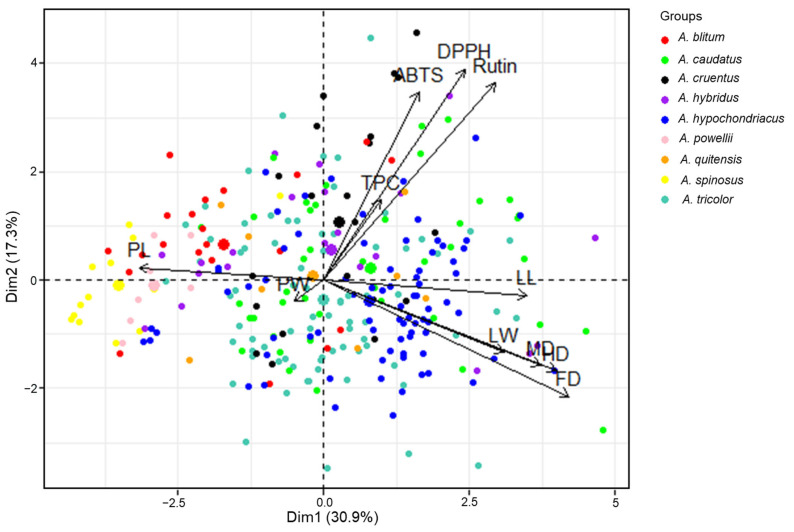
Principal component biplot for *Amaranthus* species based on their agricultural traits, phytochemical content, and antioxidant activities using the entire dataset. TPC = total phenolic content, DPPH = DPPH radical scavenging activity, ABTS = ABTS radical scavenging activity, LL = leaf length, LW = leaf width, PL = panicle length, PW = panicle width, FD = days until 50% flowering, HD = days until 50% heading, MD = days until maturity.

PCA of the agricultural traits, phytochemical contents, and antioxidant activities of the 289 accessions was performed, which yielded 11 principal components (PCs). PCs 1–4 (Table S2) had eigenvalues >1, and explained 72.62% of the total variance (30.91%, 17.30%, 13.59%, and 10.82%, respectively); therefore, the score and loading plots computed from PC1 and PC2 were used to analyze the distributions of, and associations between, agricultural traits, biochemical contents, and antioxidant activities of amaranth genotypes. The PCA showed the rough distribution of amaranth genotypes according to species. This distribution could occur if the genotypes of species differentially impacted the levels of diverse classes of agricultural characteristics, phytochemical contents, and antioxidant activities (Figure 3). PL (18.12%), PW (16.37%), MD (14.21%), LL (12.45%), and FD (10.16%) made the largest contributions to the variance that was explained by PC1. The main contributors to the variance explained by PC2 were DPPH (27.78%), rutin (24.24%), and ABTS (22.03%). The associations between the contributions observed in the PCA were consistent with the correlations derived from the Pearson’s correlation analysis. According to the score plot, *A. spinosus* and *A. powellii* were distributed on the negative side of PC1. The genetic resources of *A. spinosus* and *A. powellii* had higher PL values than other species. The comparison between PC1 and PC2 revealed a distinctive aggregation between the accessions of *A. powellii* and *A. cruentus*, *A. powellii* and *A. caudatus*, *A. cruentus* and *A. spinosus*, and *A. hypochondriacus* and *A. spinosus* (Figure 3 and Figure S1). In accordance with this, Steffensen et al. [51] demonstrated that *Amaranthus* species could be distinguished by PCA, based on the polyphenol content of seeds; however, some different results were also noted in the present study. In Steffensen’s results, *A. cruentus* and *A. hypochondriacus* were distinctively grouped by PCA, and *A. hypochondriacus* displayed a higher content of phytochemicals compared with other species [51]; however, in the present study, genotypes of *A. hypochondriacus* showed a wide variation, and they were not distinct from other *Amaranthus* species after PCA. These differences could be explained in two main ways. First, the results for amaranth leaf extracts may differ from those for amaranth seed extracts. Second, the findings of this study were based on a large number of genotypes, which allowed for more comprehensive analyses and better comparisons. 

PLS-DA was performed to confirm the relationship between agricultural traits and targeted metabolomic profiles (Figure S2). The statistical parameters obtained by PLS-DA were summarized in Table S3. The correlations observed in PLS-DA were similar to the associations observed in Pearson’s correlation analysis and PCA. TPC, rutin content, DPPH, and ABTS showed positive correlations with each other. The morphological traits LL, LW, and PW exhibited positive correlations with TPC, rutin content, DPPH, and ABTS. FD, HD, and MD exhibited positive correlations with each other. In addition, *A. cruentus* had high level of rutin content, DPPH, and ABTS. *A. spinosus*, *A. powellii*, and *A. blitum* had large size of PLs. Nevertheless, slight differences were observed, PW, for instance, displayed a positive correlation between phytochemical content and antioxidant activities in PLS-DA, but showed negative correlations in PCA. This could be attributed to the different methods used in the study. Overall, PLS-DA provided additional evidence for the relationship between agricultural traits, phytochemical content, and antioxidant activities.

**Figure 4:**
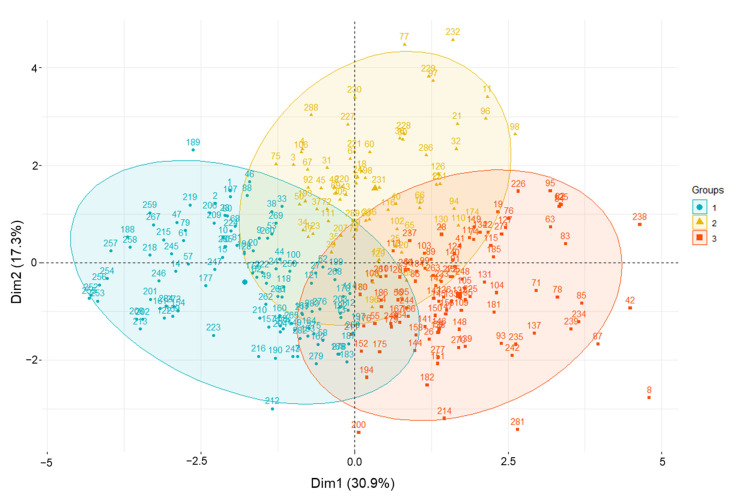
Principal component analysis biplot showing amaranth genetic resource clustering based on agricultural traits, phytochemical content, and antioxidant activities using the whole dataset.

HCPC analysis was conducted based on agricultural traits, phytochemical content, and antioxidant activities. Scatter plots of amaranth samples and loading plots are presented in Figure 4. The values for each group are summarized in Table S4, and the related amaranth genotypes used in this study are listed in Table S5. Cluster 1 contained 112 accessions which, based on PL and PW, were significantly related, and showed the lowest values of TPC, rutin, DPPH, and ABTS. Cluster 2 consisted of 74 accessions and showed the highest levels of TPC, rutin, DPPH, and ABTS. Cluster 3 included 103 accessions with high values for LL, LW, FD, HD, and MD. Based on the distance from the assigned position of each accession in the cluster to the center, the cluster analysis revealed that groups 1–3 were characterized by accession Nos. 255, 232, and 8, respectively. In cluster 2, accessions *A. cruentus* K038188 (No. 232), *A. tricolor* IT203379 (No. 77), *A. cruentus* K038185 (No. 229), *A. cruentus* IT238341 (No. 97), *A. hybridus* IT197077 (No. 11), *A. caudatus* IT238335 (No. 96), and *A. hypochondriacus* IT238342 (No. 98) were further away from their assigned position to the center, compared with other genotypes. Indeed, these accessions had high values of TPC, rutin content, DPPH, and ABTS, and thus, they could be good medical resources and potential donors for breeding programs for the development of better varieties.

## 4. Conclusions

This study demonstrated the genotypic diversity in a large population of amaranth accessions, across nine species, based on their agricultural traits, phytochemical content, and antioxidant activities. The findings of the present study suggest that some agricultural traits, including leaf color, flower color, and seed coat color, could indicate the phytochemical content and antioxidant activities of amaranth leaves. In particular, yellow-flowered amaranth genotypes could be important because of the high biochemical content level and antioxidant capacity. Among the *Amaranthus* species, *A. cruentus* had the greatest leaf length and width, and *A. spinosus* developed the highest panicle length. These genetic advantages could be considered when breeding and developing better varieties. On the other hand, *A. caudatus* accessions displayed the highest average TPC, and *A. hybridus* accessions exhibited the highest average values of ABTS. In particular, *A. cruentus* accessions could be good genetic resources since they recorded the highest average level of rutin content and DPPH radical scavenging activity. This species also displayed the second-highest level of ABTS. Overall, seven accessions of amaranth, K038188, IT203379, K038185, IT238341, IT197077, IT238335, and IT238342, were determined as being good medical resources, given the high phytochemical content and antioxidant activities in the leaves of these plants. Our study could provide important context for the development of new varieties with high phytochemical content and antioxidant activity levels. 

## Figures and Tables

**Table 1:** Basic statistics of agricultural traits, the phytochemical content, and antioxidant activities of 289 *Amaranthus* accessions.

Character	Maximum	Minimum	Mean	SD	CV (%)
TPC (µg·GAE/mg·DE)	958.19	159.62	456.05	165.35	36.26
Rutin (mg/g)	42.30	0.12	10.06	8.54	84.89
DPPH (µg·TE/mg·DE)	49.22	1.03	20.24	9.39	46.39
ABTS (µg·TE/mg·DE)	449.61	75.20	200.73	50.99	25.40
LL (cm)	32.16	6.84	21.00	6.20	29.52
LW (cm)	21.33	3.00	11.73	3.65	31.12
PL (cm)	29.10	0.66	6.26	5.51	88.02
PW (cm)	6.16	0.50	1.04	0.70	67.31
FD (day)	108.00	35.00	56.37	13.19	23.40
HD (day)	125.00	45.00	71.22	11.34	15.92
MD (day)	162.00	54.00	97.82	23.46	23.98

TPC: total phenolic content; DPPH: DPPH radical scavenging activity; ABTS: ABTS radical scavenging activity; LL: leaf length; LW: leaf width; PL: panicle length; PW: panicle width; FD: days until 50% flowering; HD: days until 50% heading; MD: days until maturity; SD: standard deviation; CV: coefficient of variation.

**Table 2:** Comparison of agricultural traits of nine *Amaranthus* species.

Scientific Name	No. Acc	Values	LL (cm)	LW (cm)	PL (cm)	PW (cm)	FD (day)	HD (day)	MD (day)
*A. blitum*	21	Range	6.8–30.0	3.5–18.4	1.8–22.8	0.5–1.5	37–62	45–69	77–87
		Mean	14.38 ^cd^	8.87 ^bc^	6.54 ^bcd^	0.97 ^ab^	44.10 ^bc^	62.19 ^b^	81.10 ^b^
		CV (%)	57.51	51.86	67.13	27.84	16.87	9.71	4.66
*A. caudatus*	43	Range	13.7–29.8	7.9–19.5	1.0–16.8	0.5–3.0	38–108	65–125	80–162
		Mean	22.05 ^ab^	12.75 ^a^	4.13 ^d^	0.91 ^b^	60.74 ^a^	74.19 ^a^	101.00 ^ab^
		CV (%)	19.00	22.82	75.54	58.24	22.79	17.81	27.35
*A. cruentus*	22	Range	16.3–32.2	6.1–21.3	2.2–19.2	0.5–4.9	37–76	63–77	78–133
		Mean	25.65 ^a^	13.30 ^a^	8.06 ^bcd^	1.64 ^a^	52.64 ^abc^	66.55 ^ab^	87.18 ^b^
		CV (%)	13.80	28.05	59.93	80.49	16.47	5.44	16.57
*A. hybridus*	21	Range	10.3–29.5	5.8–16.3	1.5–21.0	0.5–1.6	37–86	65–101	78–145
		Mean	21.90 ^ab^	11.22 ^ab^	8.54 ^bc^	0.86 ^b^	55.86 ^ab^	72.71 ^a^	96.24 ^b^
		CV (%)	26.26	28.15	76.00	38.37	29.47	18.43	24.89
*A. hypochondriacus*	77	Range	8.8–30.3	6.3–19.8	1.0–29.1	0.5–3.0	35–98	52–118	77–146
		Mean	24.49 ^a^	12.38 ^a^	5.00 ^cd^	0.93 ^b^	60.08 ^a^	74.05 ^a^	113.36 ^a^
		CV (%)	15.19	14.14	120.00	49.46	19.64	15.23	21.65
*A. powellii*	7	Range	8.2–16.5	4.9–10.4	6.7–18.1	0.5–2.0	37–48	58–65	78–86
		Mean	11.67^cd^	7.00 ^bc^	13.07 ^ab^	0.99 ^ab^	41.86 ^bc^	61.43 ^b^	82.86 ^b^
		CV (%)	23.91	31.71	28.62	50.51	10.27	5.39	4.80
*A. quitensis*	8	Range	8.9–23.5	7.2–13.5	2.8–14.4	0.5–2.6	38–73	63–79	78–133
		Mean	18.03 ^bc^	10.90 ^ab^	6.86 ^bcd^	1.09 ^ab^	55.50 ^abc^	70.25 ^ab^	107.63 ^ab^
		CV (%)	27.90	19.82	68.66	58.72	24.47	9.37	24.56
*A. spinosus*	13	Range	7.7–21.0	4.5–9.5	5.6–25.8	0.5–1.6	38–67	58–66	77–86
		Mean	10.30 ^d^	5.53 ^c^	15.48 ^a^	0.95 ^ab^	40.69 ^c^	59.46 ^b^	79.15 ^b^
		CV (%)	34.66	24.23	32.24	28.42	19.86	4.88	4.35
*A. tricolor*	77	Range	7.0–32.1	3.0–19.2	0.7–21.0	0.5–6.2	37–108	58–113	54–162
		Mean	20.15 ^b^	12.55 ^a^	5.31 ^cd^	1.13 ^ab^	58.82 ^a^	73.09 ^a^	92.04 ^b^
		CV (%)	25.46	32.03	79.10	76.99	20.16	14.90	18.70
*p*-Value			***	***	***	**	***	***	***

LL: leaf length; LW: leaf width; PL: panicle length; PW: panicle width; FD: days until 50% flowering; HD: days until 50% heading; MD: days until maturity; CV: coefficient of variation. Values within a column with different letters are significantly different (*p* < 0.05). **, *** represent significant at *p* < 0.01, 0.001, respectively.

**Table 3:** Comparison of the total phenolic content (TPC), rutin content, and DPPH and ABTS antioxidant activities of nine *Amaranthus* species.

Scientific Name	No. Acc	Values	TPC (µg·GAE/mg·DE)	Rutin (mg/g)	DPPH(µg·TE/mg·DE)	ABTS (µg·TE/mg·DE)
*A. blitum*	21	Range	204.75–958.19	0.29–27.52	4.90–37.87	100.95–327.52
		Mean	438.00 ^ab^	7.37 ^b^	21.53 ^ab^	198.09 ^abcd^
		CV (%)	43.30	100.14	39.39	26.62
*A. caudatus*	43	Range	238.02–958.19	0.19–42.30	5.98–48.75	123.62–449.61
		Mean	494.27 ^a^	12.6 ^ab^	22.45 ^a^	216.22 ^abc^
		CV (%)	35.71	76.35	49.49	31.20
*A. cruentus*	22	Range	220.23–727.25	4.74–38.80	8.09–49.22	140.05–309.64
		Mean	468.7 ^ab^	17.88 ^a^	24.34 ^a^	228.08 ^ab^
		CV (%)	36.97	62.42	46.06	19.84
*A. hybridus*	21	Range	239.01–692.33	0.82–33.79	6.51–34.15	155.46–336.63
		Mean	454.88 ^ab^	12.71 ^ab^	20.78 ^ab^	236.97 ^a^
		CV (%)	29.43	63.02	36.57	20.28
*A. hypochondriacus*	77	Range	218.26–941.39	0.37–31.11	3.59–41.74	105.40–291.94
		Mean	473.84 ^ab^	11.03 ^b^	18.5 ^ab^	196.11 ^bcd^
		CV (%)	35.78	55.12	43.24	20.63
*A. powellii*	7	Range	176.09–453.48	3.68–12.60	7.28–20.53	92.80–304.71
		Mean	277.18 ^b^	5.68 ^b^	12.19 ^ab^	184.30 ^cd^
		CV (%)	33.32	56.51	34.21	37.03
*A. quitensis*	8	Range	234.07–580.65	1.96–16.88	8.12–35.82	122.00–252.35
		Mean	450.76 ^ab^	8.43 ^b^	21.84 ^ab^	209.35 ^abcd^
		CV (%)	30.39	56.58	40.43	20.65
*A. spinosus*	13	Range	239.01–647.86	0.61–21.70	5.11–27.79	75.20–212.76
		Mean	399.28 ^ab^	3.74 ^b^	11.62 ^b^	160.84 ^d^
		CV (%)	31.96	150.80	58.52	23.69
*A. tricolor*	77	Range	159.62–775.68	0.12–40.42	1.03–47.39	96.97–272.61
		Mean	444.93 ^ab^	7.08 ^b^	21.11 ^ab^	187.05 ^cd^
		CV (%)	35.73	118.64	44.10	22.65
*p*-Value			·	***	***	***

TPC: total phenolic content; DPPH: DPPH radical scavenging activity; ABTS: ABTS radical scavenging activity; CV: coefficient of variation. Values within a column with different letters are significantly different (*p* < 0.05). · and *** represent significant at *p* < 0.1, 0.001, respectively.

**Table 4:** Pearson’s correlation coefficients of agricultural traits, total phenolic content, rutin content, and DPPH and ABTS antioxidant capacities.

Variables	TPC	Rutin	DPPH	ABTS	LL	LW	PL	PW	FD	HD
Rutin	0.25 ***									
DPPH	0.22 ***	0.68 ***								
ABTS	0.01	0.45 ***	0.44 ***							
LL	0.05	0.40 ***	0.11	0.15 **						
LW	−0.01	0.18 **	0.03	0.00	0.75 ***					
PL	−0.02	−0.16 **	−0.23 ***	−0.16 **	−0.25 ***	−0.25 ***				
PW	0.05	0.02	−0.06	−0.09	0.08	0.15 *	0.43 ***			
FD	0.10	0.13 *	0.12 *	0.01	0.33 ***	0.40 ***	−0.43 ***	−0.02		
HD	0.06	0.19 **	0.17 **	0.07	0.26 ***	0.28 ***	−0.31 ***	−0.03	0.80 ***	
MD	0.08	0.18 **	0.12*	0.03	0.30 ***	0.19 **	−0.37 ***	−0.05	0.63 ***	0.62 ***

TPC: total phenolic content; DPPH: DPPH radical scavenging activity; ABTS: ABTS radical scavenging activity; LL: leaf length; LW: leaf width; PL: panicle length; PW: panicle width; FD: days until 50% flowering; HD: days until 50% heading; MD: days until maturity. *, **, *** represent significant at *p* < 0.05, 0.01, 0.001, respectively.

## Data Availability

Data is contained within the article and Supplementary Materials.

## Supplementary Materials

The following supporting information can be downloaded at: https://www.mdpi.com/article/10.3390/plants11131758/s1. Table S1: Variations of biochemical contents and antioxidant activities in 289 *Amaranthus* accessions according to seed coat, flower, and leaf colors; Table S2: Principal component analysis of the phytochemicals, antioxidant activities, and agricultural traits of 289 accessions in nine *Amaranthus* species, with eigenvalues and individual and cumulative contributions of variables in the first five principal components; Table S3: Statistical parameters achieved by PLS-DA; Table S4: Average cluster values of agricultural traits, phytochemicals, and antioxidant activities of 289 accessions of *Amaranthus*; Table S5: List of 289 Amaranthus genotypes in this study; Figure S1: Principal component biplot for *Amaranthus* species based on their agricultural traits, phytochemical content, and antioxidant activities using the entire dataset; Figure S2: PLS discriminant analysis between agricultural traits, and phytochemical content and antioxidant activities using the entire dataset.
