# Supplementary material for: Evaluation of Agricultural Traits, Phytochemical Contents, and Antioxidant Activities in the Leaves of Amaranth Accessions of Nine Species
Source: Plants (Basel). 2022 Jul 1;11(13):1758. doi: 10.3390/plants11131758 (PMC9269144; doi:10.3390/plants11131758)
Supplement: Supplementary file 1 [file plants-11-01758-s001.zip › plants-1760790 - Supplementary.pdf]

Table S1. Variations of biochemical contents and antioxidant activities in 289 Amaranth accessions according to seed coat, flower, and leaf colors.

**Morphological characters**		**Values**	**TPC (μg·GAE/mg·DE)**	**Rutin (mg/g)**	**DPPH (μg·TE/mg·DE)**	**ABTS (μg·TE/mg·DE)**
Seed coat color	Black	Range	159.62–958.19	0.12–40.42	3.34–49.22	75.20–449.61
		Mean	457.44^{a}	8.65^{b}	19.61^{a}	198.09^{a}
		CV (%)	38.68	93.47	47.45	26.32
	Brown	Range	238.02–744.05	1.14–42.30	11.25–39.48	139.62–255.41
		Mean	460.16^{a}	14.25^{a}	23.77^{a}	190.61^{a}
		CV (%)	29.05	74.35	36.13	17.09
	Red	Range	508.83–738.78	10.11–13.91	18.17–35.67	203.05–263.07
		Mean	623.81^{a}	12.01^{ab}	26.92^{a}	233.06^{a}
		CV (%)	26.07	22.37	45.97	18.21
	Yellow	Range	204.75–718.36	0.75–33.79	1.03–48.75	96.97–351.45
		Mean	444.32^{a}	13.57^{a}	21.09^{a}	212.01^{a}
		CV (%)	28.99	60.31	46.02	23.94
Flower color	Brown	Range	176.09–941.39	0.61–31.58	4.90–39.48	100.95–449.61
		Mean	429.19^{ab}	9.22^{a}	17.71^{b}	192.76^{ab}
		CV (%)	39.41	105.22	57.03	36.55
	Green	Range	159.62–958.19	0.13–40.42	3.34–48.75	75.20–351.45
		Mean	468.25^{ab}	9.33^{a}	20.07^{ab}	192.12^{ab}
		CV (%)	35.78	82.04	44.02	22.27
	Red	Range	198.82–958.19	0.12–42.30	1.03–44.57	92.80–360.05
		Mean	427.48^{b}	10.39^{a}	20.20^{ab}	212.27^{a}
		CV (%)	36.73	81.34	44.55	25.37
	Yellow	Range	244.61–727.25	1.52–38.80	4.43–49.22	128.64–281.63
		Mean	533.18^{a}	14.08^{a}	24.56^{a}	214.36^{a}
		CV (%)	29.39	80.03	49.08	21.29
Leaf color	Red variegated green	Range	159.62–958.19	0.12–21.70	3.34–32.53	103.10–360.05
		Mean	443.29^{a}	5.77^{c}	15.47^{b}	200.64^{a}
		CV (%)	40.90	99.82	47.47	29.68
	Dark green	Range	253.18–698.92	0.81–33.69	6.94–44.57	107.44–351.45
		Mean	451.48^{a}	14.22^{a}	21.73^{ab}	215.02^{a}
		CV (%)	30.27	59.06	47.17	24.12
	Green	Range	176.09–958.19	0.37–40.42	1.03–48.75	75.20–298.15
		Mean	469.36^{a}	11.02^{ab}	20.70^{ab}	196.00^{a}
		CV (%)	36.07	75.23	45.89	22.17
	Light green	Range	221.55–861.67	0.19–38.80	7.28–49.22	97.11–284.10
		Mean	483.26^{a}	8.21^{bc}	22.65^{a}	190.05^{a}
		CV (%)	36.94	111.29	43.66	27.24
	Red	Range	212.00–744.05	0.17–42.30	4.90–38.61	100.95–449.61
		Mean	402.00^{a}	8.29^{bc}	20.23^{ab}	215.36^{a}
		CV (%)	34.33	113.76	42.73	30.05
Seed coat color		*P*-value	NS	***	NS	NS
Flower color			*	·	·	**
Leaf color			NS	***	*	·

TPC: total polyphenol content; DPPH: DPPH radical scavenging activity; ABTS: ABTS radical scavenging activity; CV: coefficient of variation;

Values in the same column marked by different superscript letters are significantly different (p<0.05);

NS, ·, *, **, *** represent not significant or significant at P < 0.1, 0.05, 0.01, 0.001, respectively.

Table S2. Principal component analysis of the phytochemicals, antioxidant activities, and agricultural traits of 289 accessions in nine *Amaranthus* species, with eigenvalues and individual and cumulative contributions of variables in the first five principal components.

**Variable**	**PC1**	**PC2**	**PC3**	**PC4**	**PC5**
TPC	1.01	3.98	0.24	24.36	56.60
Rutin	8.90	24.24	2.23	0.28	0.03
DPPH	5.98	27.78	0.50	1.47	0.43
ABTS	2.74	22.03	0.33	2.70	15.74
LL	12.45	0.14	19.15	11.66	2.11
LW	9.82	3.30	21.24	11.91	2.65
PL	18.12	8.46	1.15	4.09	0.68
PW	16.37	5.15	2.16	8.05	5.79
FD	10.16	0.10	14.50	12.12	5.55
HD	0.24	0.29	35.50	17.44	8.79
MD	14.21	4.53	3.02	5.92	1.63
Eigenvalue	3.40	1.90	1.49	1.19	0.92
Variability (%)	30.91	17.30	13.59	10.82	8.40
Cumulative (%)	30.91	48.21	61.79	72.62	81.01

TPC: total phenolic content; DPPH: DPPH radical scavenging activity; ABTS: ABTS radical scavenging activity; LL: leaf length; LW: leaf width; PL: panicle length; PW: panicle width; FD: days to 50% flowering; HD: days to 50% heading; MD: days to maturity.

Table S3. Statistical parameters achieved by PLS-DA.

Variable	Loadings		VIP	Groups	Q2	
	t1	t2			t1	t2
TPC	-0.1502	0.0496	0.4221	*A. blitum*	0.0647	-0.0050
Rutin	-0.2151	0.4874	1.0703	*A. caudatus*	0.0243	-0.0071
DPPH	-0.1282	0.2986	0.6505	*A. cruentus*	0.0014	0.1013
ABTS	-0.1329	0.4526	0.9081	*A. hybridus*	-0.0093	-0.0001
LL	-0.5309	0.2179	1.5096	*A. hypochondriacus*	0.0735	0.0149
LW	-0.4095	0.118	1.1448	*A. powellii*	0.0517	-0.0014
PL	0.3355	0.1849	0.9905	*A. quitensis*	-0.0034	-0.0041
PW	0.0008	0.3332	0.6165	*A. spinosus*	0.1675	0.0009
FD	-0.3641	-0.2343	1.0989	*A. tricolor*	-0.0057	-0.0062
HD	-0.3063	-0.2592	0.9773			
MD	-0.3294	-0.3713	1.1469			
**Component**	**R2X**	**R2X(cum)**	**R2Y**	**R2Y(cum)**		
t1	0.3035	0.3035	0.0512	0.0512		
t2	0.1601	0.4636	0.0231	0.0743		

TPC: total phenolic content; DPPH: DPPH radical scavenging activity; ABTS: ABTS radical scavenging activity; LL: leaf length; LW: leaf width; PL: panicle length; PW: panicle width; FD: days to 50% flowering; HD: days to 50% heading; MD: days to maturity; VIP: variable importance for projection; Q2: quality of loo cross-validation; R2X: Fraction of X variation modeled in that component; R2Y: Fraction of Y variation modeled in that component; cum: cumulative.

Table S4. Average cluster values of agricultural traits, phytochemicals, and antioxidant activities of 289 accessions of *Amaranthus*.

**Group**	**No. Acc.**	**TPC (µg·GAE/mg·DE)**	**Rutin (mg/g)**	**DPPH (µg·TE/mg·DE)**	**ABTS (µg·TE/mg·DE)**	**LL (cm)**
1	112	417.70 ± 182.16^{b}	3.69 ± 2.87^{c}	14.39 ± 7.26^{c}	176.54 ± 44.98^{c}	16.79 ± 6.45^{b}
2	74	497.86 ± 148.50^{a}	18.22 ± 8.61^{a}	27.39 ± 7.86^{a}	237.89 ± 52.27^{a}	23.84 ± 4.44^{a}
3	103	467.70 ± 149.27^{ab}	11.12 ± 7.07^{b}	21.47 ± 8.45^{b}	200.33 ± 39.23^{b}	23.56 ± 4.15^{a}
P-value		**	***	***	***	***
	LW (cm)	PL (cm)	PW (cm)	FD (day)	HD (day)	MD (day)
1	9.93 ± 4.23^{b}	9.27 ± 6.19^{a}	1.15 ± 0.72^{a}	48.20 ± 9.01^{b}	64.60 ± 5.30^{b}	83.48 ± 7.31^{b}
2	12.66 ± 3.38^{a}	6.03 ± 4.79^{b}	1.03 ± 0.69^{a}	51.24 ± 7.90^{b}	67.00 ± 4.27^{b}	83.78 ± 9.23^{b}
3	13.02 ± 2.04^{a}	3.20 ± 2.87^{c}	0.93 ± 0.68^{a}	68.93 ± 10.32^{a}	81.45 ± 12.37^{a}	123.50 ± 20.00^{a}
P-value	***	***	NS	***	***	***

TPC: total phenolic content; DPPH: DPPH radical scavenging activity; ABTS: ABTS radical scavenging activity; LL: leaf length; LW: leaf width; PL: panicle length; PW: panicle width; FD: days to 50% flowering; HD: days to 50% heading; MD: days to maturity;

Values in the same column marked by different superscript letters are significantly different ($p < 0.05$);

NS, **, *** represent not significant or significant at $P < 0.01$, 0.001, respectively.

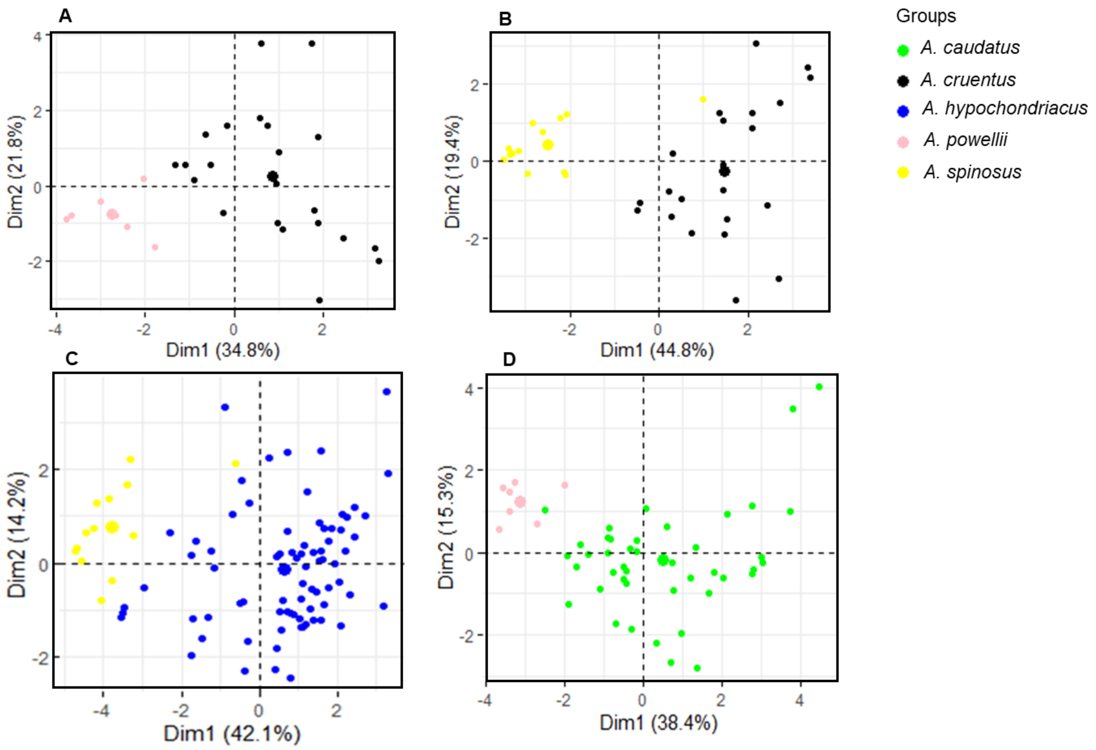

Figure S1. Principal component biplot for *Amaranthus* species based on their agricultural traits, phytochemical content, and antioxidant activities using the entire data set. (A) Loading plots of variables of *A. powellii* and *A. cruentus.* (B) Loading plots of variables of *A. spinosus* and *A. cruentus.* (C) Loading plots of variables of *A. hypochondriacus* and *A. spinosus.* (D) Loading plots of variables of *A. powellii* and *A. caudatus.*

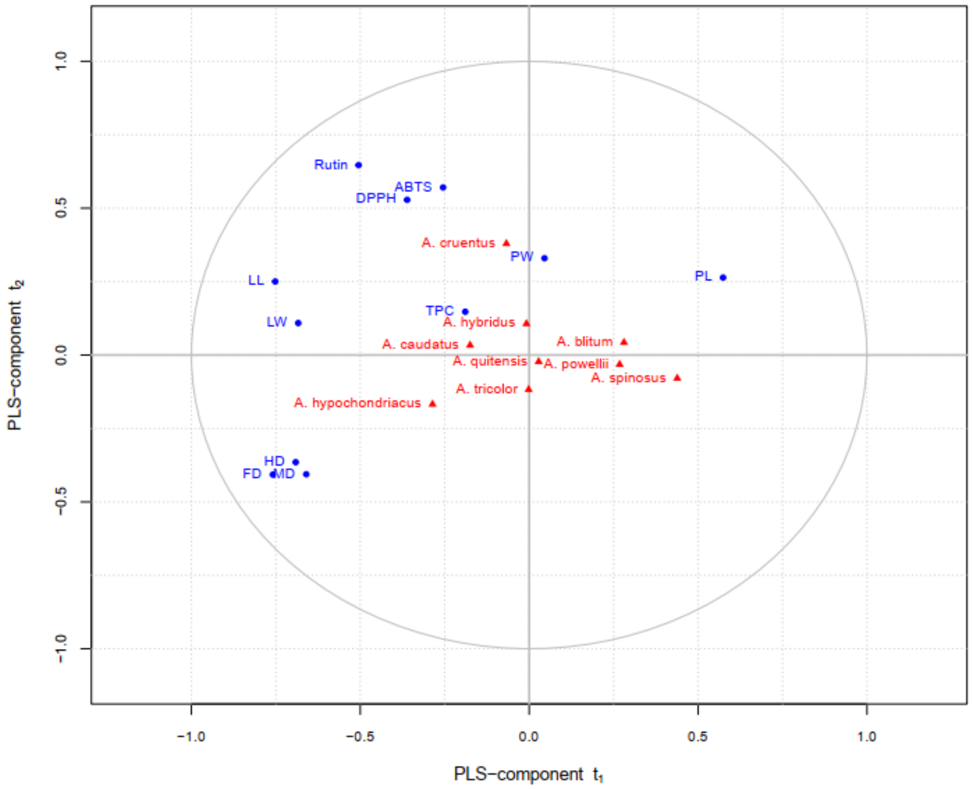

Figure S2. PLS discriminant analysis between agricultural traits, and phytochemical content and antioxidant activities using the entire data set. TPC = total phenolic content, DPPH = DPPH radical scavenging activity, ABTS = ABTS radical scavenging activity, LL = leaf length, LW = leaf width, PL = panicle length, PW = panicle width, FD = days to 50% flowering, HD = days to 50% heading, MD = days to maturity.
